# Forty five percent of the Israeli population were infected with the influenza B Victoria virus during the winter season 2015–16

**DOI:** 10.18632/oncotarget.23601

**Published:** 2017-12-22

**Authors:** Sivan Sharabi, Ravit Bassal, Nehemya Friedman, Yaron Drori, Hadar Alter, Aharona Glatman-Freedman, Musa Hindiyeh, Daniel Cohen, Ella Mendelson, Tamy Shohat, Michal Mandelboim

**Affiliations:** ^1^ Central Virology Laboratory, Ministry of Health, Chaim Sheba Medical Center, Tel-Hashomer, Ramat-Gan, Israel; ^2^ Department of Epidemiology and Preventive Medicine, School of Public Health, Sackler Faculty of Medicine, Tel-Aviv University, Tel-Aviv, Israel; ^3^ The Israel Center for Disease Control, Israel Ministry of Health, Tel-Hashomer, Ramat-Gan, Israel; ^4^ Department of Family and Community Medicine, New York Medical College, Valhalla, New York, United States

**Keywords:** influenza B, vaccine, Yamagata, Victoria

## Abstract

While infection with influenza A viruses has been extensively investigated, infections with influenza B viruses which are commonly categorized into the highly homologous Victoria and Yamagata lineages, are less studied, despite their considerable virulence. Here we used RT-PCR assays, hemagglutination inhibition assays and antibody titers to determine the levels of influenza B infection. We report of high influenza B Victoria virus prevalence in the 2015–16 winter season in Israel, affecting approximately half of the Israeli population. We further show that the Victoria B virus infected individuals of all ages and that it was present in the country throughout the entire winter season. The vaccine however included the inappropriate Yamagata virus. We propose that a quadrivalent vaccine, that includes both Yamagata and Victoria lineages, should be considered for future influenza vaccination.

## INTRODUCTION

Influenza viruses, which are members of the *Orthomyxoviridae* family, are categorized into three genera - A, B, and C [1, 2]. Infections with influenza viruses are a major cause of morbidity and mortality worldwide [3–5]. Indeed, approximately 3–5 million individuals suffer from severe illness each year with an estimated 250,000–500,000 related deaths [6]. The influenza virus undergoes rapid and significant changes that prevent the generation of long-lasting protective immunity [7, 8]. In attempt to prevent infection, the influenza vaccine composition, is re-evaluated every year. The trivalent vaccine usually contains two influenza A and one influenza B virus.

Influenza B viruses can be divided into two distinct lineages Victoria (denoted B/Victoria/2/87) and Yamagata (denoted B/Yamagata/16/88) [9–11]. The two lineages are similar and their hemagglutinin proteins show approximately 96% homology. New influenza B virus strains constantly evolve because the segmented viral genome has allowed the genetic reassortment between viruses of different lineages during co-infection [12, 13].

The two antigenically and genetically distinct influenza B viruses, have been circulating in humans since 1983. Between 1992 and 2000, Victoria lineage viruses were detected only in eastern Asia. From March to September of 2001 and during the 2001–2002 influenza season, Victoria lineage viruses were detected in several countries for the first time in a decade [14].

Several studies have demonstrated that influenza B infections can be quite dominant in a single season or across consecutive seasons [15, 16]. Yet, despite the importance of influenza B viruses, much of the published scientific literature regarding the epidemiology of influenza focused on influenza A. As a result, the global epidemiology and disease burden of influenza B viruses is still poorly understood.

The influenza trivalent vaccine given in the 2015–16 season included the A/California/7/2009 (H1N1)-like, A/Switzerland/9715293/2013 (H3N2)-like and influenza B/Phuket/3073/2013-like (B/Yamagata lineage) viruses. Evidence from the southern hemisphere and our own observations in Israel (manuscript submitted for publication), indicated that the vaccine given in the 2015–16 season was inefficient, as a significant number of individuals were infected with the influenza B and with the pandemic influenza viruses [6, 17].

The purpose of the current study was to characterize the influenza B infection during the 2015–16 season in Israel.

## RESULTS

### Influenza B virus infection prevalence between 2011 and 2016

**Figure 1:**
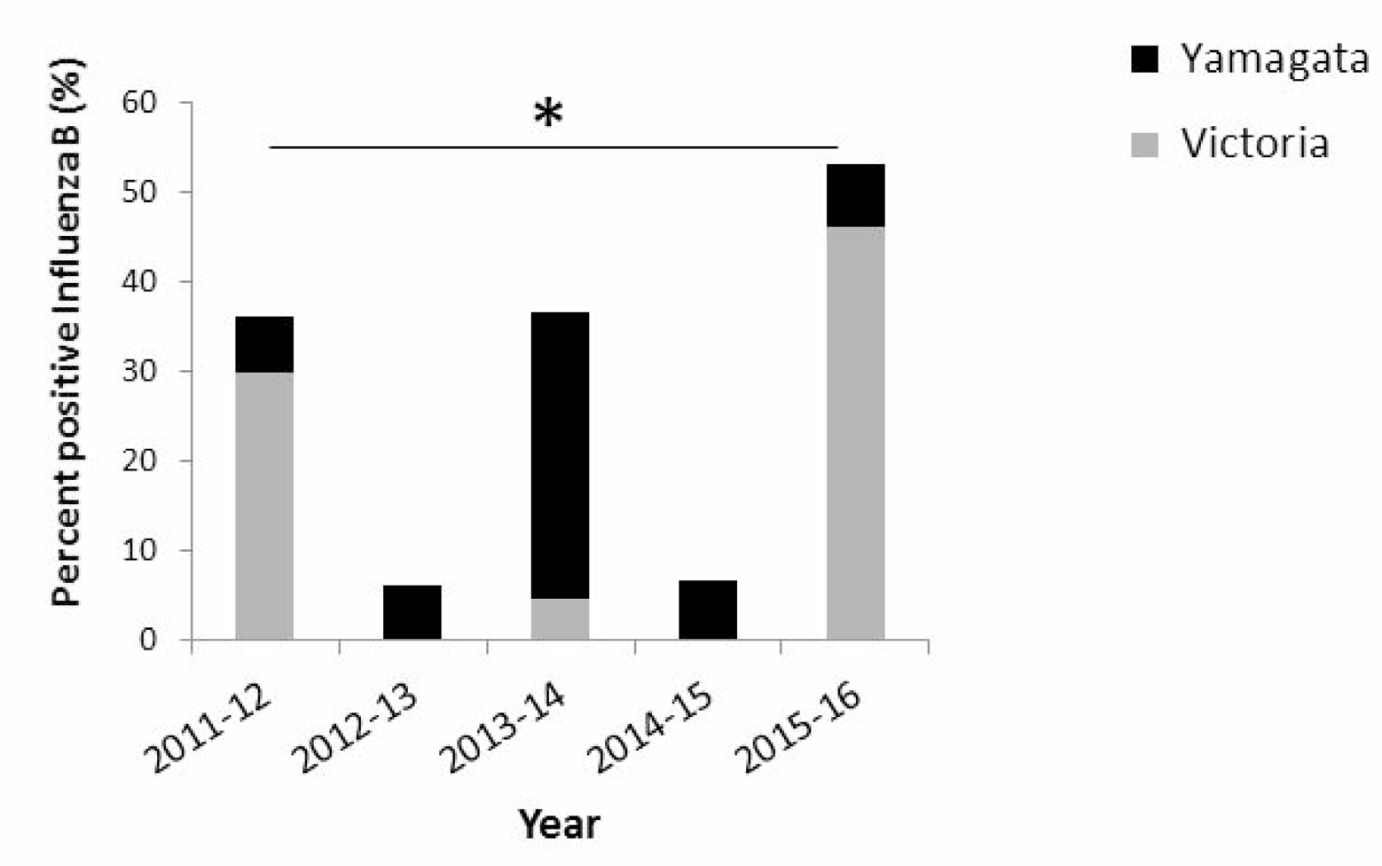
Prevalence of influenza B infections in Israel between 2011 and 2016 The presence of Yamagata and Victoria influenza B lineages, in community patients with ILI.

**Figure 2:**
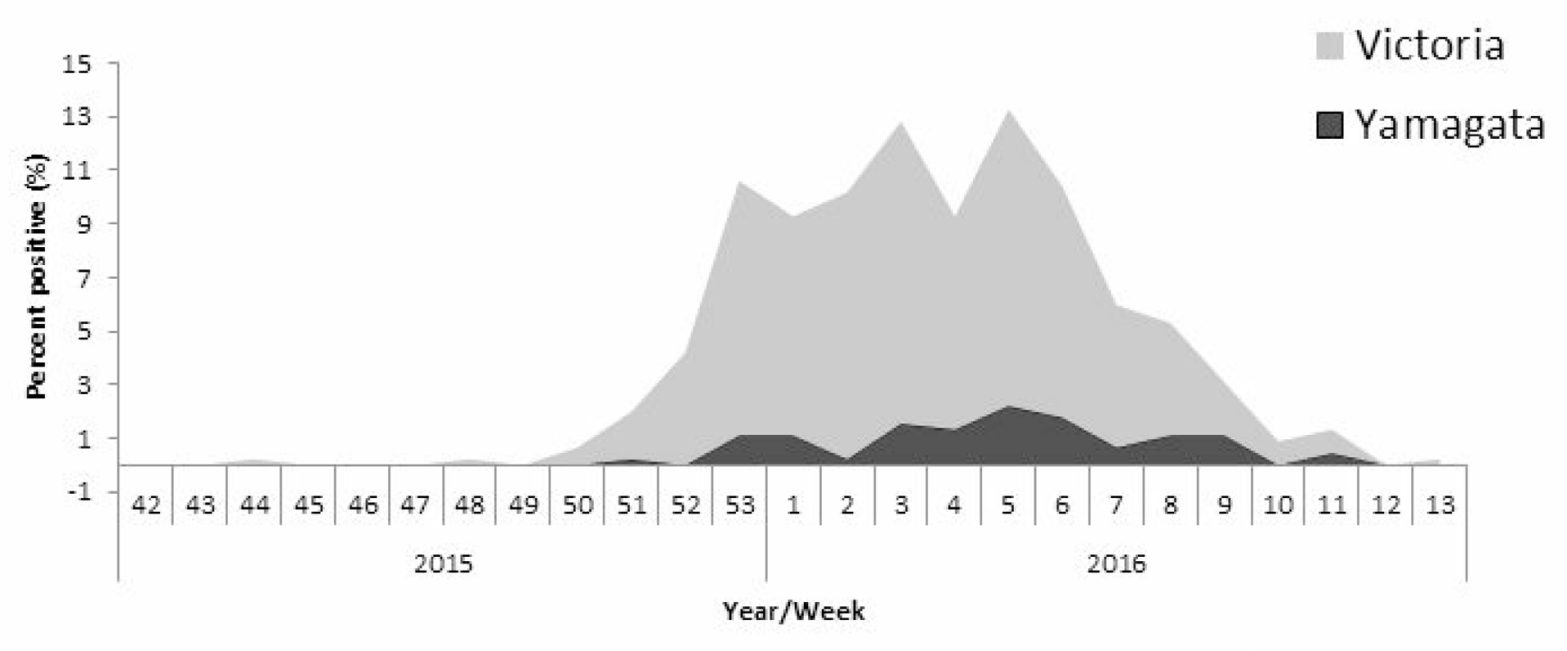
Weekly distribution of influenza B infection The graph demonstrates the weekly distribution of influenza B Yamagata and Victoria lineages detected in community ILI patients in 2015–16.

To examine the prevalence of infection by Yamagata and Victoria influenza B lineages, we determined their presence in patient samples collected between 2011 and 2016. Samples were obtained from patients suffering from Influenza-like illness (ILI), collected from sentinel clinics throughout Israel. In general, the percentages of patient samples infected with influenza B viruses varied over the years and peaks of influenza B infection were observed in the years following those in which influenza B viruses were hardly present (Figure 1). In 2011–12 and in 2013–14, 35% of all influenza infections were due to influenza B viruses. In contrast, in 2012–13 and in 2014–15, influenza B infections were detected in only 5% of the patient samples, while in the last winter season (2015–16), 50% of the influenza-infected individuals were due to influenza B (Figure 1). Interestingly, a single influenza B lineage dominated in each of the examined winter seasons. In 2011–12 and in 2015–16, the Victoria lineage was dominant, while in 2012–15, the Yamagata lineage was responsible for most influenza B infections (Figure 1). Analysis of the weekly distribution of Yamagata and Victoria influenza B viruses in the 2015–16 season showed that these viruses were detectable throughout the winter season (Figure 2).

### Sero-prevalence of antibodies to Yamagata and Victoria viruses

**Figure 3:**
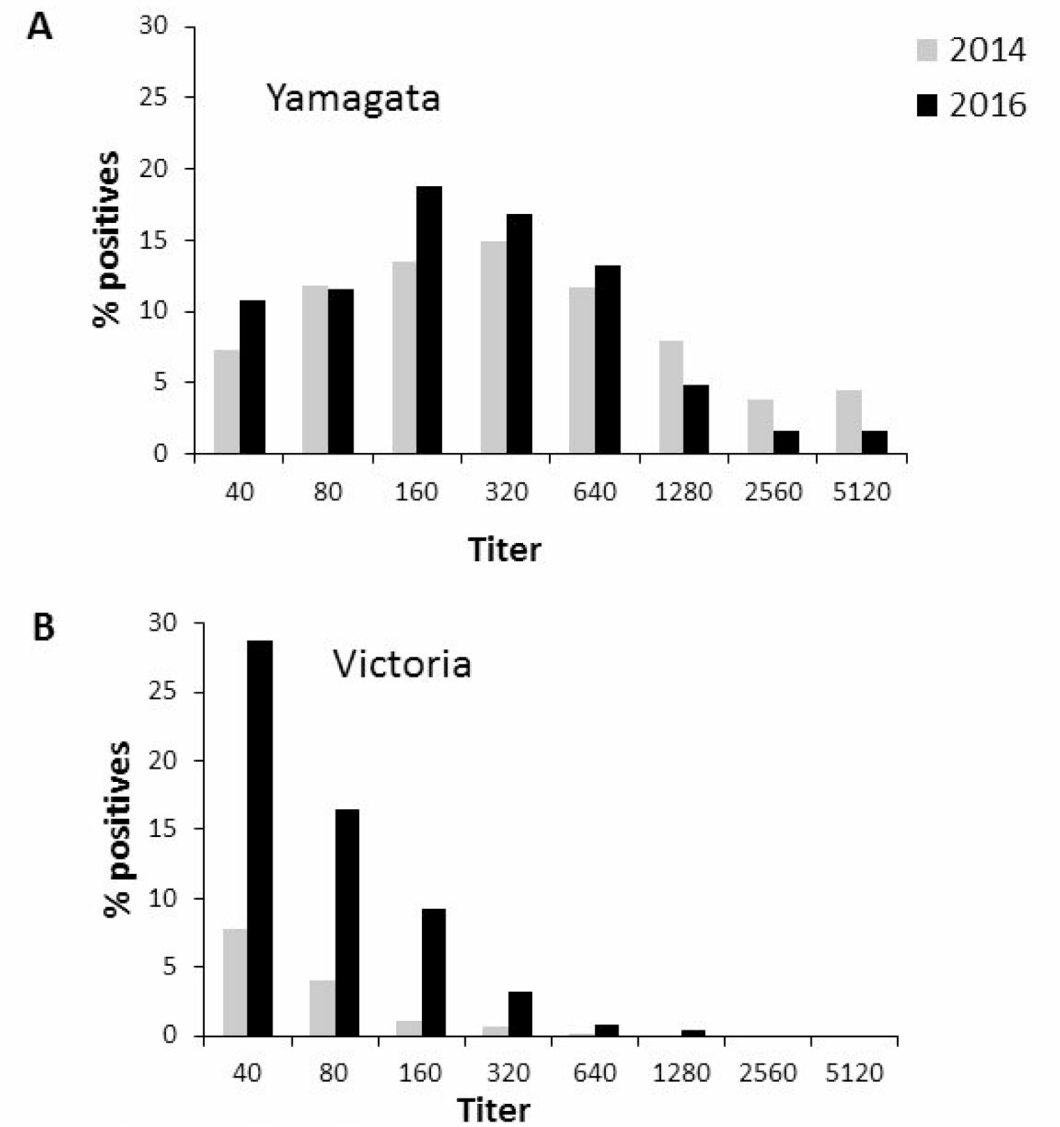
Prevalence of antibodies against Yamagata and Victoria in 2014 and 2016 Seropositivity against Yamagata (**A**) and Victoria (**B**) in 2014 and in 2016 in serum, samples stored in the Israel National Serum Bank established by the Israel Center for Disease Control.

**Figure 4:**
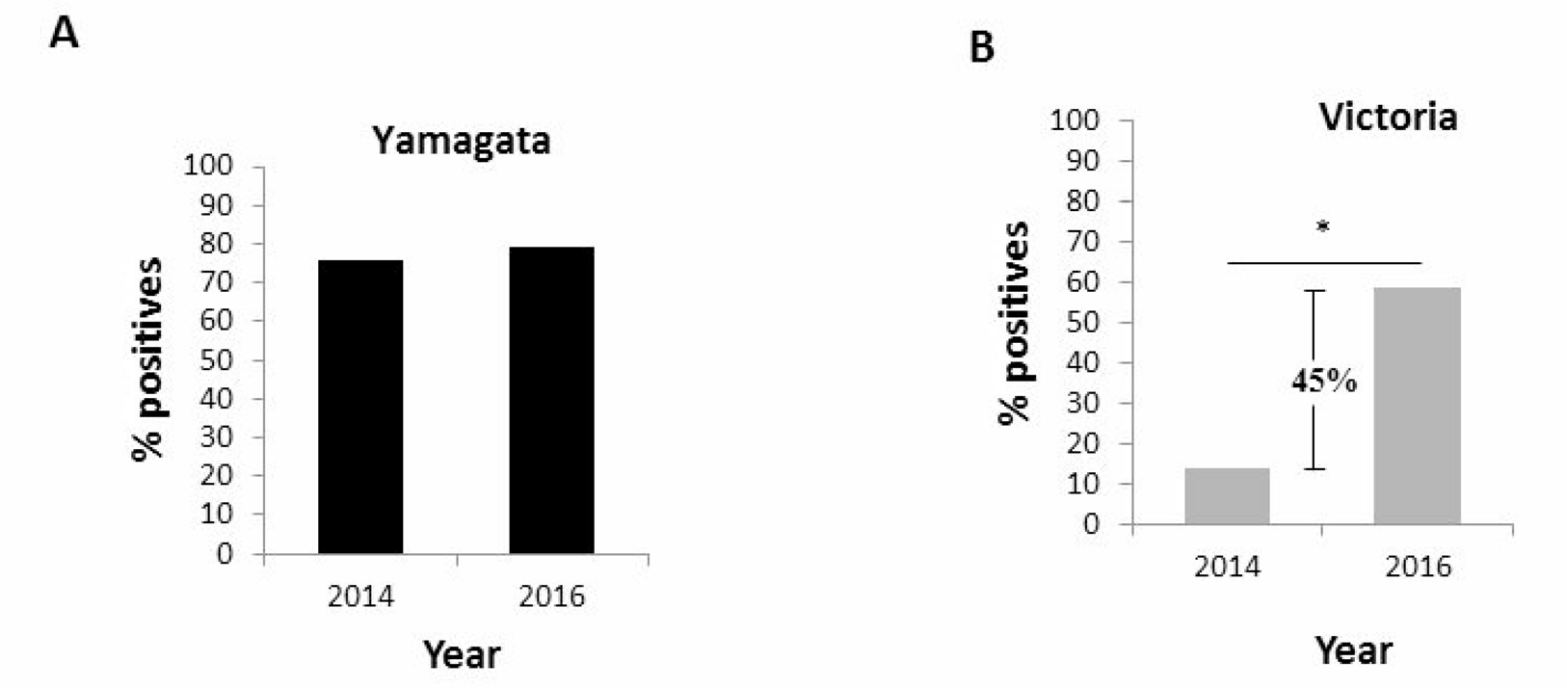
Prevalence of antibodies against Yamagata and Victoria in the entire population Presence of antibodies to Yamagata (**A**) and Victoria (**B**) influenza B viruses in 2014 and in 2016 in serum samples.

To test the extent of exposure to Yamagata and Victoria viruses in the Israeli population (both symptomatic and asymptomatic infection), we conducted a sero-prevalence study in which we examined sera obtained in 2014, when Yamagata lineage was most prevalent (Figure 1) and in 2016, when the Victoria lineage was most prevalent (Figure 1). No significant differences in Yamagata virus antibody titers in 2014 versus 2016 were noted (Figure 3A). In contrast, high antibody titers against Victoria were observed in 2016 (Figure 3B). These results prompted us to quantify the percentages of individuals having anti-Yamagata and anti-Victoria antibodies in 2014 and in 2016. Strikingly, while the percentage of individuals carrying antibodies against Yamagata was not significantly different between 2014 and 2016 (Figure 4A), 45% more individuals had anti-Victoria antibodies in 2016 as compared to 2014 (Figure 4B). This observation indicates that at least 45% of the population was infected with influenza B Victoria in the last winter season.

**Figure 5:**
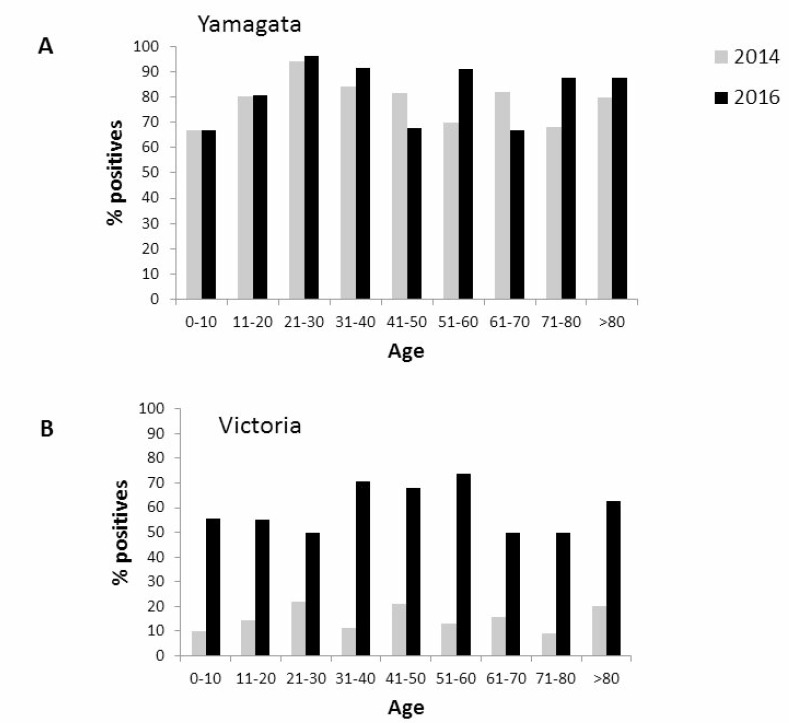
Age distribution of seropositivity against Yamagata and Victoria Age distribution of seropositivity to Yamagata (**A**) and Victoria (**B**) influenza B in 2014 and in 2016.

Finally, we analyzed the age distribution of the influenza-infected individuals. As presented in Figure 5, anti-Yamagata antibodies were detected in all age groups, in a relatively large percentages of the population examined in both 2014 and in 2016 (Figure 5A). Although anti-Victoria antibodies were detected in all age groups in both seasons, the percentages of individuals expressing anti-Victoria antibodies was higher in 2016 as compared to 2014 (Figure 5B).

## DISCUSSION

Accurate determination of the prevalence of specific lineages of circulating influenza viruses provides public health officials with information required to evaluate vaccination programs and allocate resources. The data also aid in the design of more effective vaccines and can assess whether immunity against one particular strain is effective against another influenza virus strain.

Following assessments of the 2000–02 influenza infection profiles, when the Victoria lineage became predominant worldwide [14], the World Health Organization recommended use of strain B/Hong Kong/330/2001 (Victoria lineage) in the vaccine of 2002–2003 [18, 19]. It was reported that in the southern hemisphere, some individuals were infected with the influenza/B Victoria virus. Here, we report that in the 2015–16 winter season, 45% of the Israeli population were infected with influenza B Victoria virus. In the last winter season, approximately 1.7 million individuals (20% of the population), 60.4% whom were above the age of 65, were vaccinated with the seasonal influenza vaccine in Israel. Approximately 90% of them received the trivalent vaccine, and 10% received the quadrivalent vaccine. These data, along with the reported high prevalence of Victoria virus infections, suggest that the current vaccine is not effective against the influenza B/Victoria lineage. It is possible that some individuals infected with influenza B/Victoria were asymptomatic but were involved in transmitting the infection as suggested by Hsu *et al.*, [20] and as observed for asymptomatic children infected with H1N1 in Taiwan [20, 21].

The level of cross-protection between the two B lineages is assumed to be low. Few reports have shown that infection with the Yamagata lineage influenza B virus may induce cross-lineage antibody response against the Victoria lineage as well, however, infection with the Victoria lineage fails to provide adequate protection [22–24].

Studies in the United States have shown that the frequent influenza B vaccine mismatches of recent years have been associated both with substantial increases in infection, hospitalizations and deaths [25], and with high influenza-related medical costs [26, 27]. Thus, considering the findings presented here, we recommend use of a quadrivalent influenza vaccine that contains the two influenza B lineages.

## MATERIALS AND METHODS

### Clinical samples

Nasopharyngeal and serum samples were used for our evaluation. Nasopharyngeal samples were collected as part of the community influenza surveillance conducted in collaboration with the Israel Center for Disease Control (ICDC). These samples were collected from over 20 outpatient clinics, from 1919 patients presenting with influenza-like illness (ILI) during the influenza season spanning between October 2015 and April 2016.

Serum samples (*N* = 1,018) were obtained from the Israel National Serum Bank established by the Israel Center for Disease Control. The samples were collected from individuals in 2014 and in 2016. Details regarding age groups, gender and geographical region of residence in Israel were available to the researchers and patient identity was kept anonymous. Samples from patients suffering from immunological disorders were not included in the study.

### Viral genome extraction and real-time PCR analysis (q-PCR and q-RT-PCR)

Viral genome was extracted from 500 µl of patient Nasopharyngeal samples using the NucliSENS easyMAG kit (BioMerieux, France), and eluted in 55 µl elution buffer. All samples were stored at –70°C. Sentinel samples from community patients were tested for the presence of influenza viruses (A, B, and H1N1pdm) and respiratory syncytial virus (RSV), by qRT-PCR as previously described [28, 29].

### Determination of influenza B lineages

To determine the influenza B lineage (Yamagta and Victoria), all influenza B-positive samples were subjected to a second round of qRT-PCR, performed using one set of primers and two different probes, as previously described [30]. The qRT-PCR reactions were performed in 25 µl Ambion Ag-Path Master Mix (Life Technologies, USA) using TaqMan Chemistry on the ABI 7500 instrument.

### Hemagglutination inhibition assay

All patient sera were treated with receptor destroying enzyme (RDE) (Sigma C8772, diluted 1:4), for 16 h prior to heat inactivation (30 min, 56°C). Absorption with erythrocytes was performed to remove non-specific hemagglutination, in accordance with a modified WHO protocol [31]. Serial two-fold dilutions (1:20–1:2560) of sera in 25 μl PBS were prepared in V-shaped well plates, and an equal volume of four hemagglutinin (HA) units of viral antigen was added. The mixture was then incubated at room temperature for 1 h. Fifty microliters of 0.5% chicken erythrocytes suspended in PBS, were added to the wells, and mixed by shaking the plates on a mechanical vibrator. Agglutination patterns were read after 30 min and the hemagglutination inhibition (HI) titer was defined as the reciprocal of the last dilution of serum that fully inhibited hemagglutination. The cut-off value selected for a positive result was 1:40. The influenza B antigens (B/Brisbane/60/2008- Victoria and B/Massachusetts/2/2012 –Yamagata) were supplied by the WHO.

### Statistical analysis

The Chi-square test was applied to evaluate the differences in percent positivity between the compared groups. A *p* value < 0.05 was considered statistically significant. All analyses were performed using SPSS (version 21.0.0. SPSS Inc., Chicago, IL, USA), SAS (SAS 9.1, SAS Institute Inc, Cary, NC, USA) and Excel software.

### Ethical considerations

Nasopharyngeal samples were obtained as part of influenza surveillance conducted in accordance with the Public Health Ordinance in Israel and did not necessitate informed consent. Serum was obtained from anonymous leftover diagnostic samples, and as such did not require informed consent. The institutional review board (IRB) of the Sheba Medical Center approved this research, under Helsinki protocol numbers 9750-12-SMC and 2873-15-SMC.

This work was performed in partial fulfillment of the requirements for an M.Sc. degree of Sivan Sharabi, Sackler Faculty of Medicine, Tel Aviv University, Israel.
